# Exploring the optimal impact force for chronic skeletal muscle injury induced by drop-mass technique in rats

**DOI:** 10.3389/fphys.2023.1241187

**Published:** 2023-08-09

**Authors:** Haiya Ge, Zhengming Wang, Zongrui Yang, Jinyu Shi, Jiehang Lu, Yuanyuan Wang, Zhengyan Li, Guoqing Du, Zhibi Shen, Hongsheng Zhan

**Affiliations:** ^1^ Shi’s Center of Orthopedics and Traumatology, Shuguang Hospital Affiliated to Shanghai University of Traditional Chinese Medicine, Shanghai, China; ^2^ Institute of Traumatology and Orthopedics, Shanghai Academy of Traditional Chinese Medicine, Shanghai, China

**Keywords:** chronic muscle injury, contusion, inflammation, extracellular matrix, ultrastructure

## Abstract

**Introduction:** Skeletal muscle injuries are widespread in sports, traffic accidents and natural disasters and some of them with poor prognoses can lead to chronic skeletal muscle damage in the clinic. We induced a chronic skeletal muscle injury by controlling time and contusion force using an acute blunt trauma model that will help us better comprehend the pathological features of chronic skeletal muscle injury.

**Methods:** Several levels of injury were induced by repeatedly striking in 5, 10, and 15 times the gastrocnemius muscle from the same height with 200 g weights. After injury, the markers of muscle injury were assessed at 2 and 4 weeks by serum elisa. Electron microscopy, histologic and immunohistochemical staining, and mRNA analysis were used to evaluate the ultrastructure, inflammation, extracellular matrix decomposition, and anabolism of injured muscle in 2 and 4 weeks.

**Results:** All three different kinetic energies can result in skeletal muscle injuries. However, the injured skeletal muscles of rats in each group could not recover within 2 weeks. After 4 weeks, tissue self-repair and reconstruction caused the damage induced by 5 J kinetic energy to almost return to normal. In contrast, damage induced by 10 J kinetic energy displayed slight improvement compared to that at 2 weeks. Despite this, collagen fibers on the surface of the tissue were disorganized, directionally ambiguous, and intertwined with each other. Myofilaments within the tissue were also arranged disorderly, with blurry and broken Z-lines. Damage caused by 15 J kinetic energy was the most severe and displayed no improvements at 4 weeks compared to 2 weeks. At 4 weeks, IL-1β, IL-6, Collagen I, and Collagen III, MMP2 expressions in the 10 J group were lower than those at 2 weeks, showing a tendency towards injury stabilization.

**Conclusion:** After 4 weeks of remodeling and repair, the acute skeletal muscle injury model induced by 10 J kinetic energy can stabilize pathological manifestations, inflammatory expression, and extracellular matrix synthesis and catabolism, making it an appropriate model for studying chronic skeletal muscle injuries caused by acute injury.

## Introduction

Skeletal muscle injuries are widespread in sports, traffic accidents and natural disasters, which can be classified as acute and chronic depending on etiologies (Flores et al., 2018; Wang and Zhou, 2022). Although some mild acute injuries can be effectively treated and cured, there are other acute injuries that can develop from acute to chronic due to severe damage or ineffective treatment, resulting in limited range of motion and weakness and affecting quality of people’s life (Noonan and Garrett, 1999; Greising et al., 2020). Moreover, in recent years, the incidence rate of chronic skeletal muscle injuries such as Primary injury (e.g., muscle strain caused by long-term work-related posture) and Chronic secondary muscle injury (e.g., the outcome of acute injuries) has increased year by year (Zhuang et al., 2014; Liu et al., 2018). Therefore, it is extremely important to study pathogenesis of skeletal muscle injury, understand its pathological characteristics and clinical outcomes, investigate related prevention, treatment, and rehabilitation approaches.

A good animal model is the basis for experimental research on the pathogenesis of skeletal muscle injury, and different animal models are applicable to different research purpose. At present, there are many animal models of skeletal muscle injury. The commonly used methods for the construction of acute muscle injury models include intramuscular injection of biotoxins or chemicals such as CTX, notexin (NTX), barium chloride (BaCl2), glycerol, mechanical injury such as freezing, crushing, contusion, and electrical (Hardy et al., 2016; Liu et al., 2022). However, the number of chronic injury models is far away from the acute one and the most of them are based of some disease such as duchenne muscular dystrophy (Fiore et al., 2020). Dystrophin-deficient mdx mice is the most widely used animal model for research of chronic injury (Magown et al., 2015; Bronisz-Budzyńska et al., 2020). While this model can make skeletal muscles in state of frequent damage-healing, it cannot fully mimic the pathological changes of skeletal muscle repair after moderate and severe acute injuries.

In this study, we use the 200 g weight dropping from a height of 50 cm with different times to mimic the mild, moderate, and severe acute injury and investigate the change of injured skeletal muscle by both morphology and molecular studies in the natural process of muscle damage repair after injury at 2 weeks and 4 weeks. Finally, we identified the impact force and optimal harvesting time for rats with chronic injury from moderate acute injury.

## Materials and methods

### Animals

Adult male Sprague-Dawley rats (200 ± 20g, Charles River) were used for modeling. All procedures were performed with the approval of the Institutional Animal Care and Use Committee of Shanghai University of Traditional Chinese Medicine (PZSHTCM211129021). A total of 40 animals were used in this study and killed at 2 weeks and 4weeks after the contusion was imposed.

### Contusion and grouping

**FIGURE 1:**
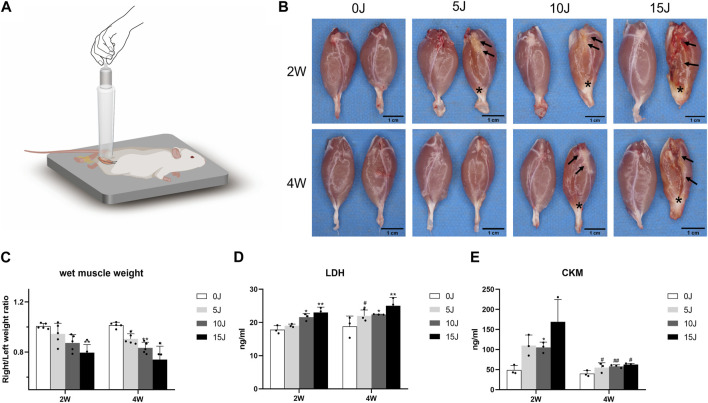
**(A)** Schematic of the drop-mass method. A 200 g cylindrical weight with a bottom surface diameter of 2.8 cm was dropped from the top of 50 cm tube vertically on the gastrocnemius muscle of the right hind limb of rats for different times to induce different degrees of muscle injury. “↑”: tissue organization; “∗”: tendo calcaneus thickening. **(B)** Gross observation of gastrocnemius muscles on the control and intervention side. **(C)** The wet weight ratio of gastrocnemius in different groups. **(D–E)** Expression of CKM and LDH in serum. Data were expressed as Mean ± SD. (n = 3–5). *p < 0.05, **p < 0.01 when compared with 0 J group at the same time point; ^#^ *p* < 0.05, ^##^ *p* < 0.01 when compared with the corresponding group at 2 weeks.

Forty rats were randomly divided into 0J, 5 J,10J, 15 J groups. Except 0 J group, other rats were placed in a prone position with the right hind limb extending and ankle dorsiflexing to 90° after anesthetized by Zoletil 50 (50 mg/kg). 200 g balancer was raised to a height of 50 cm with an acrylic guide tube and dropped onto the gastrocnemius muscle to establish the model of skeletal muscle injury (Figure 1A). Different times of strikes were used to exploring different force of the blow on rats.

According to the Work formula of Gravity:

W=m∙g∙h

W is the work done, ***m*** is the mass of the hammer, ***g*** = 9.8 N/kg (1 kg object 9.8 N under gravity), ***h*** is the distance (height) in the direction of gravity. The sum of the work done at different times of strike can be calculated in Table1.

**TABLE 1:** Blow work and their sum with different times.

Subject	Times of blowing		
	5 times (J)	10 times (J)	15 times (J)
Single Work done	1		
Sum of the Work Done	5	10	15

### Wet muscle weight

In this study, wet muscle weight was used to observe the degree of injury at different stages. Five rats in each group were killed with deeply anesthetized and then the gastrocnemius muscle were removed from both two limbs and weighed by electronic balance.

#### Electron microscopy

The ultrastructural changes in skeletal muscle were assessed using electron microscopy. The internal structure of the muscle was observed by a TEM, while the surface changes were explored by a SEM. The muscles were fixed with 2% glutaraldehyde and 1% osmic acid, dehydrated in gradient ethanol and embedded in acetone, sectioned, stained with uranyl acetate and lead citrate, and then examined via transmission electron microscopy. The same method was used to fix muscles for SEM observation, dry through critical points, and observe tissue surface changes in each group after metal coating.

### Histological analysis

The rats were sacrificed and the right gastrocnemius were removed and fixed for 2days with 4% paraformaldehyde (biosharp, BL539A). After that, samples were rinsed with running water for 1 h to remove residual paraformaldehyde. The muscle tissues were embedded in paraffin and sliced to 4 μm. Certain paraffin sections were stained with H&E staining kit (Solarbio, G1120) following the manufacturer’s instructions. The remaining paraffin sections were subjected to Sirius red staining kit (Polysciences, 24,901–250) according to instruction.

### Immunohistochemistry

The tissue sections were fixed and embedded routinely. The antigen was repaired with sodium citrate at 100°C, incubated with 3% hydrogen peroxide at room temperature for 10min, and blocked with blocking solution at 37 °C for 30min. The slices were then incubated with primary antibodies (collagenⅠ1:500, collagen Ⅲ 1:500, MMP2 1:400) overnight and secondary antibodies for 30min at 37 °C. Finally, DAB staining was performed, followed by hematoxylin counterstaining.

### Serum elisa

The levels of LDH and CKM in serum reflect the severity of chronic muscle injury. The blood samples were rapidly taken from abdominal aorta of rats then allowed to stand for 2 h, centrifuged for 10 min at 3,000 rpm to obtain serum. The expression of LDH and CKM in serum were measured by enzyme-linked immunosorbent assay kits (ELK Biotechnology, ELK5692, ELK2529) following the manufacturer’s protocols.

### Quantitative real-time PCR analysis

Total RNA was extracted from gastrocnemius muscle using Trizol Reagent (Invitrogen, 15596026CN). Reverse transcription and cDNA synthesis was performed in two-step method using the PrimeScript™ RT reagent Kit with gDNA Eraser (Takara, RR047A), according the manufacturer’s protocol. qPCR was performed using the TB Green^®^ Premix Ex Taq™ II Kit (Takara, RR820A) to amplify cDNA with standard protocol. The thermocycling conditions for RT-qPCR were 30 s at 95 °C, then 5 s at 95 °C, 30 s at 60 °C (40 cycles), finally 15 s at 95 °C, 1min at 60 °C, 15 s at 95 °C for extension. GAPDH was used as the internal control to analyze the expression of mRNA. The fold change was calculated with the -2^ΔΔ^CT method. Primer sequence presented in Table 2.

**TABLE 2:** Primer sequences used in quantitative real-time PCR.

Gene	Primer sequences
**IL-1β**	F: CTG​TGA​CTC​GTG​GGA​TGA​TG
	R: GGG​ATT​TTG​TCG​TTG​CTT​GT
**IL-6**	F: GCT​CGT​CGT​CGA​CAA​CGG​CCT​C
	R: CAA​ACA​TGA​TCT​GGG​TCA​TCT​TCT​C
**MMP2**	F: ACC​TGA​ACA​CTT​TCT​ATG​GCT​G
	R: CTT​CCG​CAT​GGT​CTC​GAT​G
**Collagen Ⅰ α1**	F: ATC​AGC​CCA​AAC​CCC​AAG​GAG​A
	R: CGC​AGG​AAG​GTC​AGC​TGG​ATA​G
**Collagen Ⅲ α1**	F: TGA​TGG​GAT​CCA​ATG​AGG​GAG​A
	R: GAG​TCT​CAT​GGC​CTT​GCG​TGT​TT
**GAPDH**	F: ATG​GGA​AGC​TGG​TCA​TCA​AC
	R: GTG​GTT​CAC​ACC​CAT​CAC​AA

### Statistical analysis

All data are represented by mean ± standard (mean ± SD). The experimental data was processed by SPSS 23.0 software (SPSS, Chicago, IL, United States). The difference between groups was analyzed by one way ANOVA and Leven’s test was used to check the homogeneity of variance. An independent samples *t*-test was used for comparison between each groups at different time points. Statistical significance was set at *p* < 0.05.

## Result

### Changes of muscle wet weight within 2 and 4 weeks after varying degrees of injury

All of the rats had right hind leg contusion injuries. Three rats were excluded from this study due to tibial fractures caused by contusion. Except for the rats with severe impairment, the other rats dispalyed normal activity after 2 and 4 weeks of modeling. Compared with the left normal gastrocnemius muscle, except for the 5 J group at 4 weeks, the gastrocnemius tissue of the other injury groups showed different degrees of destruction at 2 weeks and 4 weeks, which were characterized by tissue organization and tendo calcaneus thickening. (Figure 1B). The wet weight of the gastrocnemius muscle on the affected side decreased after 2 weeks in the 5J and 10 J group, but there was no statistical significance. The wet weight of the muscle in the 15 J groups was significantly lower than in the control group (*p* < 0.01). After 4 weeks, the gastrocnemius muscle on the affected side of rats with varying degrees of injury showed atrophy (*p* < 0.05, *p* < 0.01) (Figure 1C).

### Changes of muscle damage markers within 2 and 4 weeks after varying degrees of injury

The serum CKM expression of the rats in the 10 J groups was higher compared to the control group after 2 weeks (*p* < 0.05, *p* < 0.01), however, there was no significant difference observed in the concentration of CKM in the 5J and 15 J injured group when compared to the control group. After 4 weeks, CKM expression in each injured group was not statistically significant when compared with the control group, but the corresponding 4-week injured groups had significantly lower serum CKM expression than the 2-week injured groups (Figure 1E). The LDH concentration was higher in the 10J and 15 J groups than in the control group after 2 weeks (*p* < 0.05, *p* < 0.01), with no statistically significant difference observed between the 5 J and control groups. All injured groups had a higher expression of LDH than the control group after 4 weeks (*p* < 0.05, *p* < 0.01); however, no significant difference in expression levels of LDH was found between the 2- and 4-week groups except 5 J group (Figure 1D).

### Pathological changes of muscle within 2 and 4 weeks after varying degrees of injury

**FIGURE 2:**
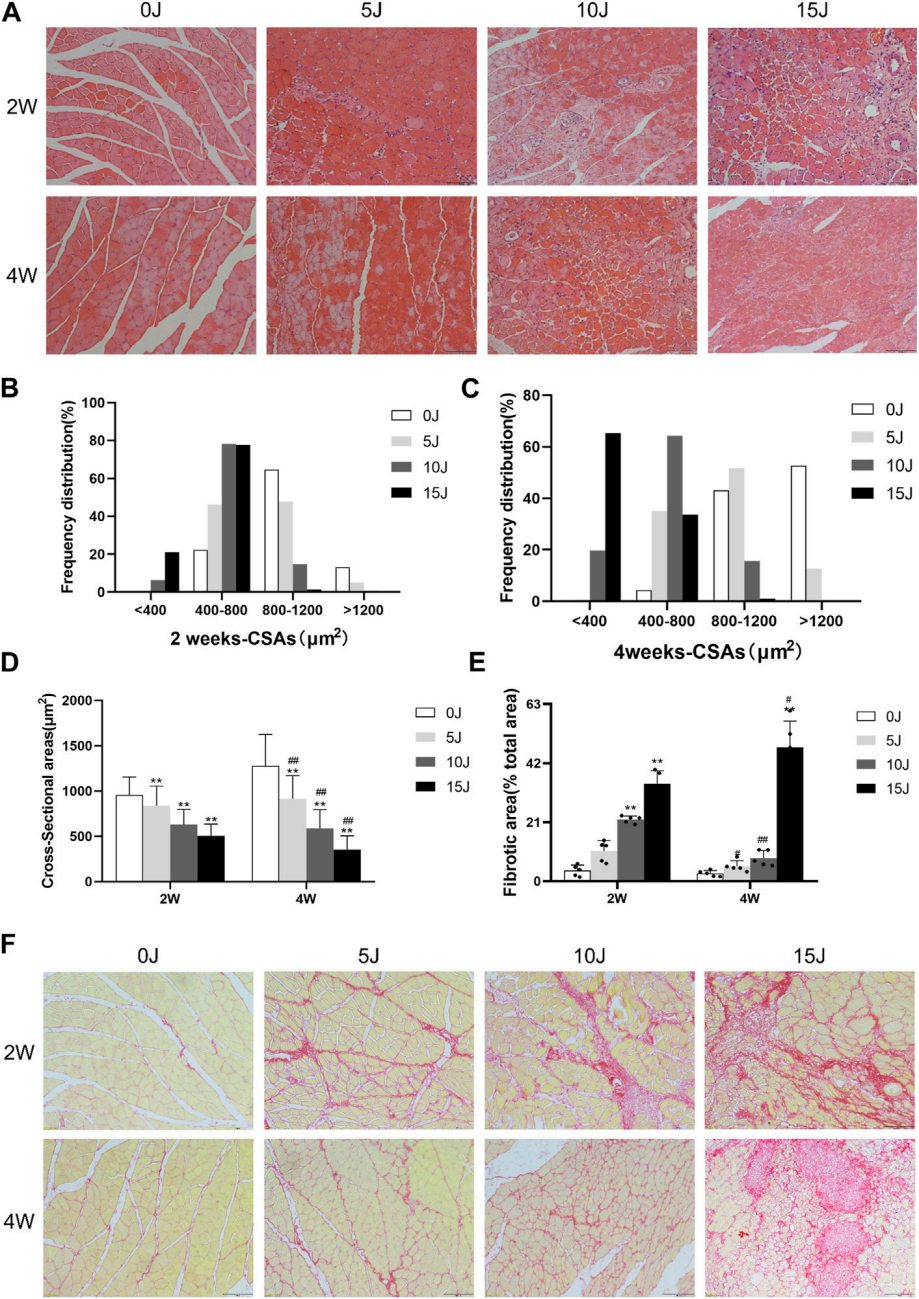
**(A)** H&E staining of the muscle. **(B–C)** Frequency distributions of myofiber cross-sectional area at 2 weeks and 4 weeks. **(D)** CSA of myofibers. **(E)** Quantification of fibrosis area. **(F)** Picrosirius red staining for fibrotic area. Scale bars = 100 µm. Data were expressed as Mean ± SD. (n = 3–5). *p < 0.05, **p < 0.01 when compared with 0 J group at the same time point; ^#^ *p* < 0.05, ^##^ *p* < 0.01 when compared with the corresponding group at 2 weeks.

The results of HE staining revealed that 2 weeks after injury, the muscle fibers of the gastrocnemius muscles in the 5J, 10J, and 15 J groups had varying degrees of injury. The muscle fibers in the control group have a higher proportion of 800–1200 μm^2^ and were polygonal and regular in shape. The sarcolemma had good integrity and the muscle nuclei were evenly dispersed without hyperplasia. Muscle fibers in the 5 J group were circular and varied in size, with inflammatory cell infiltration in some places. The CSA of single muscle fiber was largely concentrated in 400–800μm^2^ and 800–1200 μm^2^. The muscle fibers of the 10 J group had inflammatory cell infiltration, proliferative blood vessels, and dissolving muscle fibers increased, and the CSA of single muscle fiber was reduced, with the majority of it in 400–800 μm^2^. In the muscle fibers of the 15 J group, there was a high number of inflammatory cell infiltration and muscle fiber breakdown, and the area of a single muscle fiber was largely below 400μm^2^ and 400–800 μm^2^. After 4 weeks of regeneration, the number of inflammatory cell infiltration in the stroma decreased considerably in 5 J group. While the number of proliferative vessels was reduced in the 10 J group, the number of inflammatory cells infiltrated was increased, as did the percentage of smaller fibers (<400 μm^2^). A considerable number of muscle fibers were dissolved in the 15 J group, there was still inflammatory cell infiltration, and the CSA of single muscle fiber was typically less than 400 μm^2^ (Figures 2A–C). The results of total muscle fiber area also showed a decrease in each injury group compared to the control group at the same period (*p* < 0.01) (Figure 2D). In addition, from the different periods after the same degree of injury, except for a slight increase in the area of muscle fibers in group 5 J at 4 weeks, the area of muscle fibers in the other injury groups decreased significantly compared with the corresponding injury groups at 2 weeks (*p* < 0.01). This indicated that the muscle fibers of the 5 J group tended to be normal after self-repair; the muscle fibers of the 10 J injury group were partially repaired but still impaired; and the muscle fibers of the 15 J injury group were more damaged than repaired because the muscle fibers were too severely destroyed.

### Changes of collagen content within 2 and 4 weeks after varying degrees of muscle injury

Sirius red staining was used to examine the changes in collagen content in the gastrocnemius muscle of rats during the recovery process at 2 and 4 weeks following injury. At 2 weeks following injury, the collagen content in skeletal muscle of the 10J, and 15 J groups was considerably higher than that of the control group (*p* < 0.01). The collagen content of the 5 J group was not significantly different from that of the control group at 4 weeks, but it was lower compared to that of the 5 J group at 2 weeks (*p* < 0.05), indicating that the pathological changes of the 5 J group were gradually repaired after 4 weeks of repair. The fibrotic areas of 15 groups were considerably increased when compared to the control group at the same period (*p* < 0.01). The collagen content in the 10 J group was lower after 4 weeks than it was at 2 weeks (*p* < 0.01). However, as compared to the same group at 2 weeks, the collagen content of the 15 J group at 4 weeks still showed an upward trend (*p* < 0.01), indicating that applying 15 J of force to muscle was too severe to self-repair (Figures 2E,F).

### Ultrastructural changes of muscle within 2 and 4 weeks after varying degrees of injury

**FIGURE 3:**
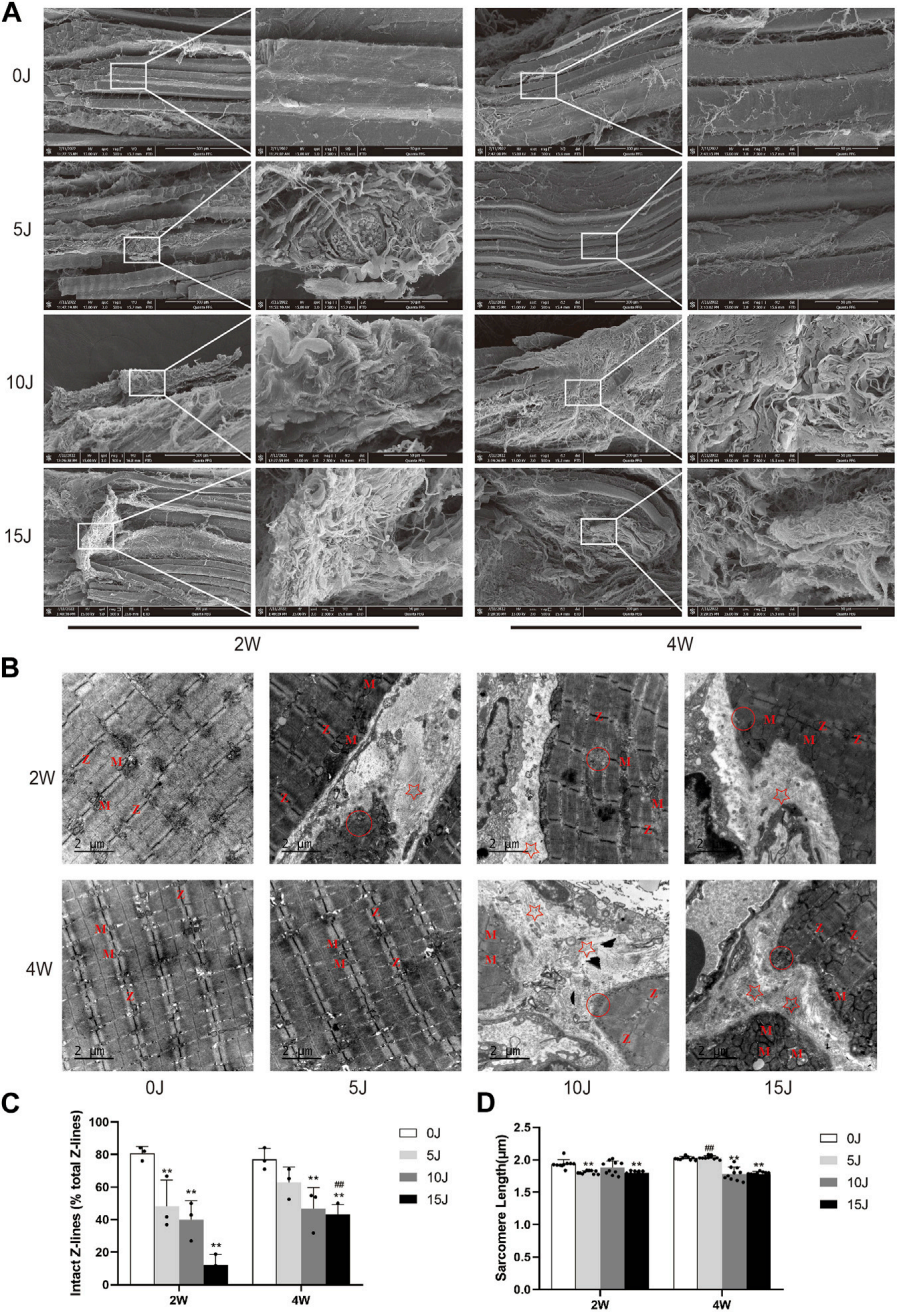
**(A)** Ultrastructure of muscle surface detected by scanning electron microscopy. **(B)** Ultrastructure of muscle tissue detected by electron microscopy. “Z”: z-line; “M”: mitochondria; “☆”: Deposited collagen fibers; “○“: myofilament dissolving. **(C)** The number of intact Z-lines. **(D)** The length of single sarcomere. Data were expressed as Mean ± SD. (n = 3–5). **p < 0.01 when compared with 0 J group at the same time point; ^##^ *p* < 0.01 when compared with the corresponding group at 2 weeks.

The alterations in muscle fiber and ECM structure were investigated using SEM at 2 and 4 weeks following varying degrees of impairment. At week 2, control group muscle fibers displayed a neat, clear arrangement with minimal collagen cables present on the surface (Figure 3A, 2W-0J). Conversely, in the 5 J group, the texture of muscle fibers was disorganized, directionally ambiguous, nonuniform in thickness, and exhibited localized collagen fiber deposition with relatively chaotic arrangement (Figure 3A, 2W-5J). The 10J and 15 J groups experienced varying degrees of thickening of muscle fiber membranes, along with widened gaps between myofibrils. Collagen fibers were observed to be disorganized, oriented in random directions, and entangled with each other (Figure 3A, 2W-10J, 2W-15J). After 4 weeks, the amount of muscle fibers and collagen fibers in the 5 J group approached normal levels (Figure 3A, 4W-5J), while the 10J and 15 J groups continued to exhibit significant collagen fiber deposition compared to week 2, with the latter group experiencing more severe deposition (Figure 3A, 4W-10J, 4W-15J).

**FIGURE 4:**
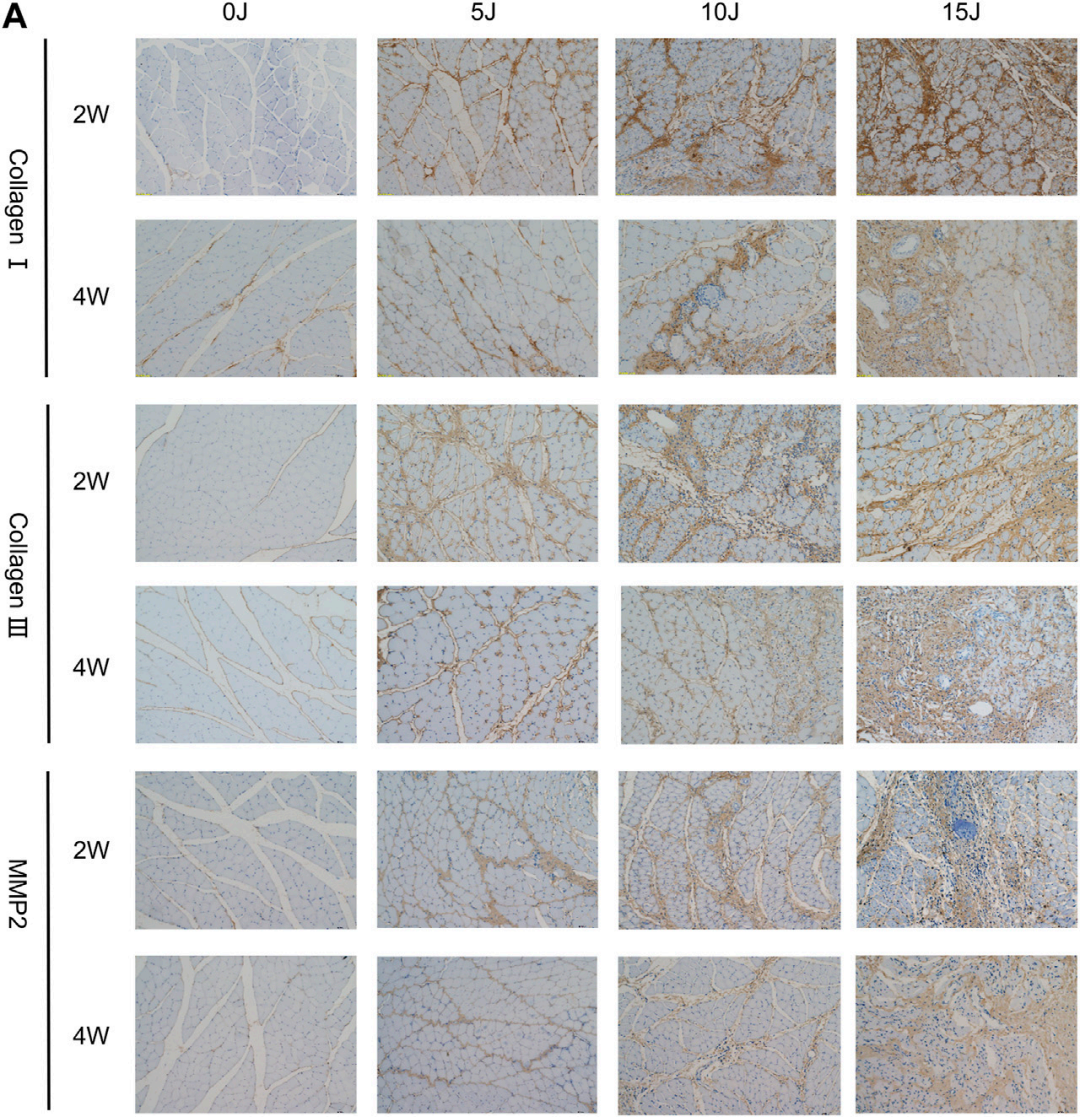
Immunohistochemical staining of collagen I, collagen III and MMP2. Scale bars = 100 µm.

**FIGURE 5:**
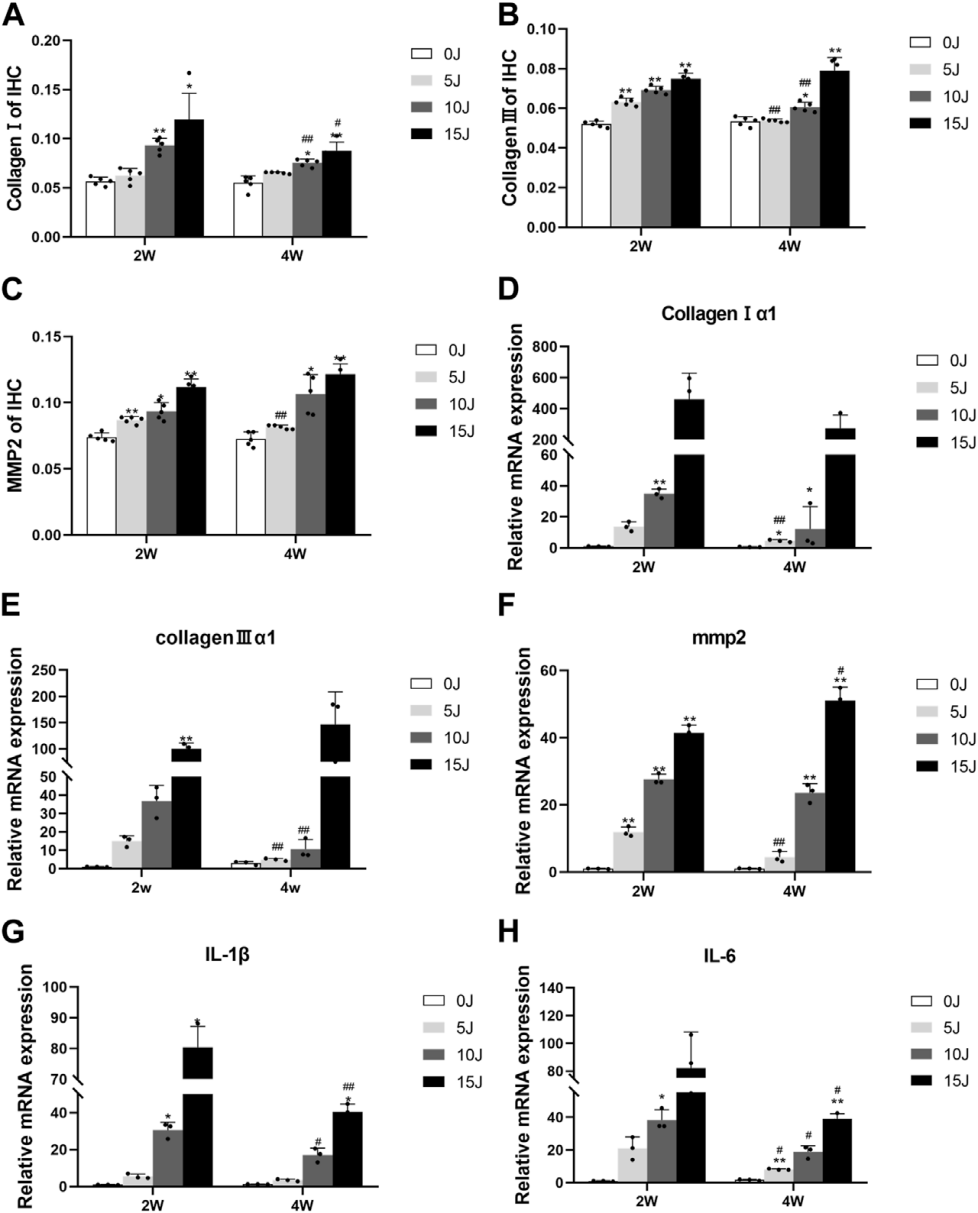
**(A–C)** Immunohistochemical analysis of collagen I, collagen III and MMP2 protein expression. **(D–F)** The mRNA expression of collagen I, collagen III and MMP2. **(G–H)** The mRNA expression of IL-1β, IL-6. Data were expressed as Mean ± SD. (n = 3–5). *p < 0.05, **p < 0.01 when compared with 0 J group at the same time point; ^#^ *p* < 0.05, ^##^ *p* < 0.01 when compared with the corresponding group at 2 weeks.

Transmission electron microscopy was used to examine the ultrastructure of skeletal muscular cells after 2 and 4 weeks of injury. The boundaries of the bright and dark bands of the myofibrils were clear at 2 weeks, the Z-line was arranged clearly and neatly, and mitochondria were orderly arranged on both sides of the Z-line (Figure 3B, 2W-0J). But there was varying degrees of damage of myofibrils in the 5J, 10J, and 15 J groups. While the light and dark bands were neatly arranged and the mitochondria were still arranged in pairs on both sides of the Z line in the 5 J group, the number of complete Z line decreased and the length of sarcomeres shortened (*p* < 0.01) (Figure 3B, 2W-5J, 3C, D). The bright and dark bands of other sarcomeres were relatively clear in the 10 J group, the Z-line was fuzzy and disruption, and the number of mitochondria was reduced and swollen (Figure 3B, 2W-10J). The light and dark bands and Z-lines of other sarcomeres were blurred in the 15 J group, and the mitochondria were clearly swollen (Figures 3B, 2–15J). In addition, sarcomere length and the number of intact Z-lines of the 15 J groups were shortened or decreased when compared with the control group (*p* < 0.01). (Figures 3C,D). At 4 weeks, the ultrastructure of muscle fibers in the 5 J injury group tended to be normal, with clear and orderly light and dark bands and Z-lines, and mitochondria arranged on both sides of the Z-line (Figure 3B, 4W-5J). Despite the presence of dissolved myofibrils in the 10 J injury group, the rest of the sarcomere structure was normal (Figure 3B, 4W-10J). Not only did the dissolved myofibrils persist in the 15 J injury group, but so did the disordered Z-line, blurred light and dark bands, and mitochondrial swelling (Figure 3B, 4W-15J). At 4 weeks, the sarcomere length was significantly shorter and the number of intact Z-lines was reduced in all injury groups except the 5 J group compared with the control group (*p* < 0.01). For the same degree of injury, the number of intact Z-lines in the 15 J group at 4 weeks was significantly higher than that at 2 weeks (*p* < 0.01). Moreover, sarcomere length in the 5 J group at 4 weeks was restored compared with that at 2 weeks (*p* < 0.01). (Figures 3C,D).

### Changes of extracellular matrix within 2 and 4 weeks after varying degrees of muscle injury

Immunohistochemistry was used to evaluate the expressions of collagen I, collagen III, and mmp-2 in skeletal muscle in order to investigate the balance of extracellular matrix catabolism and anabolism during tissue healing following injury. After 2 weeks, the protein expressions of collagen III and mmp-2 in each damaged group were significantly higher than in the control group (*p* < 0.01). While collagen I increased in the 10J and 15 J groups (*p* < 0.05, *p* < 0.01), they were not statistically significant when compared to the control group in the 5 J group. At 4 weeks, the expressions of collagen I, collagen III, and mmp2 in the 5 J group had no statistical significance when compared to the control group, but expression in the 10J and 15 J injury groups was still higher compared to the control group (*p* < 0.05, *p* < 0.01). This demonstrated that after 4 weeks of repair, extracellular matrix synthesis and catabolism tended to be normal level in group of 5 J. Furthermore, following 2 weeks of healing, the expression of collagen III and mmp-2 in the 5 J group at week 4 was lower than that at week 2 (*p* < 0.01). Collagen I and Collagen III levels were lower in the 10 J injury group compared to the 2 weeks (*p* < 0.01) and the Collagen I content of the 15 J injured group was lower too (*p* < 0.05) (Figure 4; Figures 5A–C).

### Changes in relative expression levels of extracellular matrix and inflammatory factors within 2 and 4 weeks after varying degrees of muscle injury

Two weeks post-injury, the mRNA levels of collagen Ⅰ, Ⅲ, and mmp2 were significantly higher in all injury groups than those in the control group (*p* < 0.01), except for collagen Ⅰ, Ⅲ in the 5 J group, collagen Ⅰ in 15 J group and collagen Ⅲ in 10 J group. At 4 weeks post-injury, collagen Ⅰ mRNA levels remained elevated in the 5J and 10 J groups, while mmp2 mRNA levels were still increased in the 10J and 15 J groups compared to the control group (*p* < 0.01). The expression of collagen Ⅰ, Collagen Ⅲ and mmp2 in the 5 J group, as well as collagen Ⅲ in the 10 J group, decreased significantly at 4 weeks (*p* < 0.05, *p* < 0.01) compared to 2 weeks, whereas the mmp2 mRNA level was still higher in the 15 J group (*p* < 0.05). (Figures 5D–F).

We also assessed the inflammatory response in the injured tissue at 2 and 4 weeks. Results indicated that IL-1β and IL-6 mRNA levels in the 5 J group did not significantly differ from those in the control group at either time point. However, the expressions of IL-1β and IL-6 mRNA in the 10J and 15 J groups were higher than those in the control group (*p* < 0.05, *p* < 0.01), and their levels at 4 weeks were lower than those at 2 weeks (*p* < 0.05, *p* < 0.01), implying that these groups had already entered the chronic inflammatory phase by 4 weeks post-injury (Figures 5G,H).

## Discussion

Skeletal muscle is the most vulnerable tissue in the human body due to its widespread distribution (Qazi et al., 2015). In our daily life, contusion is the most common type of mechanical injury, which occurs in traffic accidents and sports when the muscle is injured by a rapid, powerful, and non-penetrating force (Canale et al., 1981; Dement and Lipscomb, 1999). Clinically, muscle injuries can be classified into three grades (mild, moderate, and severe) according to the severity of contusions: The mild injury presents a limited degree of muscle fiber damage with slight edema and little or very slight impairment of strength and mobility; the moderate injury shows a large area impairment of muscle fibers with strength decreasing significantly; A severe injury is one that affects the entire muscle cross-section as well as the surrounding muscle tissue, resulting in a complete loss of muscle function (Pollock et al., 2014; Mcaleer et al., 2022). Although early and efficient therapy can assist to lessen clinical symptoms in all acute injuries, treatment of the subacute and chronic stage is equally critical, especially in some moderate and severe skeletal muscle injuries (Dubois and Esculier, 2020).

Since the prognosis of some subacute and chronic stage of acute injury remains poor and there are no effective treatment options so far, it is still worthy of continued investigation. Currently, drop-mass technique, which has high maneuverability, is the most frequently used method to establish the model of acute mechanical injury in animal experiments (Souza and Gottfried, 2013). However, because these studies used varied mass weights and heights, the injuries differed in terms of impact load and severity. For example, the lowest impact kinetic energy estimated was 0.48 J while the highest reached at 6.5072 J for the same type of animal (Wistar adult) and muscle (gastrocnemius) (Singh et al., 2017; Hartmann et al., 2020). Furthermore, it is a model of acute injury, researchers pay more attention to pathological alterations in the acute rather than chronic phase, therefore some experiments intervene immediately after successful modeling, while others begin several hours later (Singh et al., 2017). Here, we have observed for the first time the ultrastructural and pathological alterations of skeletal muscle in subacute and chronic phase after different degrees of traumatic muscle injury. Besides, we also examined the expression of injury-related factors, inflammation-related factors and extracellular matrix catabolism and anabolic factors in the sub-acute and chronic stages of injury after different degrees of injury.

In previous study, the pathological process of acute injury can be divided into the acute (within 7 days), subacute stage (7–14days) and chronic stage (>28days) (Kim et al., 2012). Moreover, one research studied the expression of many genes, e.g., specific markers of immune cells, regeneration regulatory factors, inflammatory cytokines at different time points after high-energy blunt injury and found that these genes almost returned to normal at 14 days post-injury. This indicates that skeletal muscle requires at least 2 weeks to recover after injury (Xiao et al., 2016). Since the current study concentrated mainly with the pathological alterations in the subacute and chronic phases following acute injury, the pathological changes were assessed at 2 and 4 weeks after modeling. Besides, Singh et al. (Singh et al., 2017) has found the injured muscle restore as normal tissue structure after 28 days with 6 J Kinetic energy impact injury. Based on this, we assigned a mild damage force of 5J, a moderate damage force of 10J, and a severe damage force of 15 J.

CKM and LDH are two commonly used biomarkers for the diagnosis of muscle damage (Brancaccio et al., 2010; Hsu et al., 2020). In previous study, LDH was found to peak around 24 h after injury and subsequently return to baseline levels within 3–6 days (Lippi et al., 2018). In this sense, we observed a significant increase in CKM expression in each of the injured groups. However, our results showed that the expression of CKM at 2 weeks post-injury was twice that of 4 weeks post-injury, indicating that CKM expression remained elevated for a longer time period in cases of severe injury. In contrast, LDH levels only showed a slight increase 3 h after injury and returned to normal within 24 h, which is consistent with our results (Brancaccio et al., 2010; Lippi et al., 2018). It is also important to note that LDH values were not significantly different at 2 weeks compared to 4 weeks post-injury. This may reflect the limitations of using LDH as a biomarker for the diagnosis of subacute and chronic muscle damage. Given the sensitivity of CKM expression and its gradual return to normal, it may be a more reliable indicator of muscle damage severity and recovery.

Inflammation is the initial response to acute injury, characterized by a rapid increase in blood flow, cellular infiltration, and release of pro-inflammatory mediators. To date, the effect of inflammation on tissue repair remains debatable. Some scholars hold the view that inflammation is a key process that eliminates injured cells, coordinates regeneration responses, and restores tissue homeostasis, whereas other scholars believe that it is a detrimental process associated with tissue damage, pain, and delayed damage (Toumi and Best, 2003; Chazaud, 2016). In this sense, the HE staining revealed a significant amount of inflammatory infiltration and vascular proliferation in the moderate and severe injury groups, both at 2 and 4 weeks post-injury. We also examined the expression of IL-1β and IL-6 and detect that the severer muscular impairment, the higher the concentration of local inflammatory factors and the levels of IL-1β and IL-6 declined over time as the duration except mild injured group. Furthermore, some previous study also showed that inflammation has identified as a critical biological process contributing to muscle loss (Tuttle et al., 2020). Our own research also supports this notion, as we have observed a decrease in both muscle wet weight and cross-sectional area in all damage groups. While there was no significant difference between the 2-week and 4-week time points, it is clear that inflammation plays a crucial role in contributing to muscle loss.

In addition to inflammation, the catabolism and anabolism of ECM have a considerable influence on skeletal muscle repair and remodeling (Mahdy, 2019). The ultrastructure in our study revealed that collagen fibers increased in quantity and displayed disordered arrangement at each injury group. Additionally, with TEM and SEM, fibroblasts and deposited collagen could be observed around the injured muscle fibers in skeletal muscle tissue after injury. During the tissue repair phase, collagen expression increases approximately 2 weeks post-injury to support structural integrity and stabilize the affected area. Subsequently, during the tissue remodeling process, ECM catabolic enzymes facilitate tissue remodeling via collagenase digestion (Filippin et al., 2009). However, in cases of severe injuries, this imbalance results in excessive collagen synthesis, which leads to tissue fibrosis (Csapo et al., 2020). Our Sirius red staining results indicated that after 2 weeks, each injury group was still in the repair stage and collagen remained highly expressed. Four weeks later, the mild injury group demonstrated normalcy, while the severe injury group exhibited severe fibrosis due to excessive collagen expression. Within the skeletal muscle ECM, type I collagen and type III collagen are major factors that contribute to anabolism and MMP-2 plays a key role in catabolism within skeletal muscle tissues (Gillies and Lieber, 2011). In this sense, we investigated the expression of Col I, Col III, and MMP2 and observed findings consistent with previous morphological observations.

## Conclusion

In our research, we employed three distinct kinetic energies to generate varying degrees of chronic skeletal muscle injury. We then investigated the ultrastructure and pathological changes in tissue during the subacute and chronic stages after the onset of injury. Our results indicate that three different kinetic energies can indeed damage skeletal muscles, which cannot return to normal within 14 days. However, after 4 weeks of repair, the skeletal muscle structure of injuries caused by 5 J kinetic energy exhibited a recovery. In contrast, injuries induced by 10J and 15 J entered into the chronic phase of repair after 4 weeks. Of the two, the injury caused by 10 J demonstrated milder ultrastructure, pathology, inflammation, and collagen expression than its 15 J counterpart, which was too severe to improve. Hence, studying the repair mechanism of skeletal muscle during the chronic phase of acute injury is more appropriate for injuries caused by 10 J.

Although this modeling methodology possesses non-invasive, adjustable, and simplistic attributes, lack of sophistication in the modeling apparatus may result in inadequate fixation of skeletal muscle injuries and uneven degrees of injury. Henceforth, forthcoming research necessitates more sophisticated instruments and experimental verification involving a larger sample size.

## Data Availability

The original contributions presented in the study are included in the article/supplementary material, further inquiries can be directed to the corresponding author.

## Abbreviations

IL-1β, Interleukin-1beta; IL-6, Interleukin-6; MMP2, Matrix metalloproteinase 2; Collagen Ⅰ, Type Ⅰ Collagen; Collagen Ⅲ, Type Ⅲ Collagen; ECM, Extracellular matrix; TEM, Transmission electron microscopy; SEM, Scanning electron microscopy; H&E, Hematoxylin-eosin; LDH, Lactate Dehydrogenase; CKM, Creatine Kinase, Muscle; CSA, Cross sectional area.
